# Correlation between acute ischaemic stroke clot length before mechanical thrombectomy and extracted clot area: Impact of thrombus size on number of passes for clot removal and final recanalization

**DOI:** 10.1177/23969873211024777

**Published:** 2021-07-07

**Authors:** Rosanna Rossi, Seán Fitzgerald, Sara M Gil, Oana M Mereuta, Andrew Douglas, Abhay Pandit, Paul Brennan, Sarah Power, Jack Alderson, Alan O’Hare, Michael Gilvarry, Ray McCarthy, Klearchos Psychogios, Georgios Magoufis, Georgios Tsivgoulis, István Szikora, Katarina Jood, Petra Redfors, Annika Nordanstig, Erik Ceder, Turgut Tatlisumak, Alexandros Rentzos, John Thornton, Karen M Doyle

**Affiliations:** 1 Department of Physiology and Galway Neuroscience Centre, School of Medicine, National University of Ireland, Galway, Ireland; 2 CÚRAM–SFI Research Centre in Medical Devices, National University of Ireland Galway, Galway, Ireland; 3 Department of Radiology, Royal College of Surgeons in Ireland, Beaumont Hospital, Dublin, Ireland; 4 Cerenovus, Galway, Ireland; 5 Metropolitan Hospital, Department of Neuroradiology, Piraeus, Greece; 6 Second Department of Neurology, National & Kapodistrian University of Athens, “Attikon” University Hospital, Athens, Greece; 7 National Institute of Clinical Neurosciences, Department of Neurointerventions, Budapest, Hungary; 8 Department of Neurology, Sahlgrenska University Hospital, Gothenburg, Sweden, and Department of Clinical Neuroscience, Institute of Neuroscience and Physiology, Sahlgrenska Academy at University of Gothenburg, Gothenburg, Sweden; 9 Department of Interventional and Diagnostic Neuroradiology, Sahlgrenska University Hospital, Gothenburg, Sweden

**Keywords:** Acute ischemic stroke, thrombectomy, computed tomography

## Abstract

**Introduction:**

We assessed the correlation between thrombus size before and after mechanical thrombectomy, measured as length by Computed Tomography Angiography/Non-Contrast Computed Tomography (CTA/NCCT) and Extracted Clot Area, ECA, respectively. We also assessed the influence of thrombus size on the number of passes required for clot removal and final recanalization outcome.

**Materials and methods:**

Acute ischaemic stroke (AIS) thrombi retrieved by mechanical thrombectomy from 500 patients and data of clot length by CTA/NCCT were collected from three hospitals in Europe. ECA was obtained by measuring the area of the extracted clot. Non-parametric tests were used for data analysis.

**Results:**

A strong positive correlation was found between clot length on CTA/NCCT and ECA (rho = 0.619,N = 500,P < 0.0001*). Vessel size influences clot length on CTA/NCCT (H2 = 98.6, P < 0.0001*) and ECA (H2 = 105.6,P < 0.0001*), but the significant correlation between CTA/NCCT length and ECA was evident in all vessels. Poorer revascularisation outcome was associated with more passes (H5 = 73.1, P < 0.0001*). More passes were required to remove longer clots (CTA/NCCT; H4 = 31.4, P < 0.0001*; ECA; H4 = 50.2, P < 0.0001*). There was no significant main association between recanalization outcome and length on CTA/NCCT or ECA, but medium sized clots (ECA 20–40 mm^2^) were associated with least passes and highest revascularisation outcome (N = 500, X^2^ = 16.2, P < 0.0001*).

**Conclusion:**

Clot length on CTA/NCCT strongly correlates with ECA. Occlusion location influences clot size. More passes are associated with poorer revascularisation outcome and bigger clots. The relationship between size and revascularisation outcome is more complex. Clots of medium ECA take less passes to remove and are associated with better recanalization outcome than both smaller and larger clots.

## Introduction

In daily practice, the occlusion location and estimate of the size of the thrombus in acute ischemic stroke (AIS) patients are important characteristics evaluated during the assessment phase of a stroke patient.^1,2^ Several ways of measuring thrombus size have been reported in the literature, in terms of volume, length or burden score.^3–6^ We evaluated thrombus size both before and after mechanical thrombectomy to investigate the correlation between these two variables. We measured thrombus size before thrombectomy as clot length on CTA/NCCT, while for thrombus size evaluation after thrombectomy we introduced a new parameter that we called Extracted Clot Area, ECA, an assessment of the area of the retrieved clot. Additionally, we interrogated the association between thrombus size and the number of passes and recanalization outcome, expressed as final mTICI score. Finally, we compared the findings in patients that were pre-treated with rtPA before mechanical thrombectomy and patients that underwent mechanical thrombectomy without thrombolysis, in order to assess the possible influence of rtPA.

## Methods

### Patient cohort and clinical data

We collected clots from 500 AIS patients as part of the RESTORE registry of acute ischemic stroke clots. The RESTORE Registry is a registry of thrombotic material extracted via mechanical thrombectomy from patients suffering from Acute Ischemic Stroke in four international stroke centres in Europe. The RESTORE Registry will account for clots collected from 1000 patients when complete. A main aim of the Registry is to analyse clots from AIS patients and correlate clot characteristics with procedural information. For this reason, patients for which no clot could be extracted are not part of the Registry and we have no information on these patients. All patients included in the study underwent mechanical thrombectomy between February 2018 and November 2019 for acute occlusion of a large artery in the anterior or posterior circulation. 44% of the patients also received intravenous thrombolysis therapy. The inclusion criteria were: patients >18 years, having undergone mechanical thrombectomy treatment for AIS and having thrombus material available for analysis.

Pertinent clinical data were collected from each patient in the form of an anonymized data abstraction form including whether the patient had been pre-treated with intravenous rtPA before mechanical thrombectomy, the approximate clot length on CTA/NCCT, the number of passes (attempts) required to remove the clot. Final reperfusion was evaluated as modified Treatment In Cerebral Ischemia (mTICI) score^7^ and assessed by anteroposterior/lateral Digital Subtraction Angiography, certified by at least two radiologists. Approval by ethics committees of each participating stroke centre and by National University of Ireland Galway research ethics committees (16-SEPT-08) were obtained before this study following the ethical standards of the Declaration of Helsinki and its amendments.^8^

### Thrombus size measurement

Thrombus length on CTA/NCCT was measured before thrombectomy in the endovascular suite at each respective hospital. Using multiphase CTA, a combination of phases was used to visualise the proximal and distal ends of the clot. Clot length was measured using CTA in 342 of the 500 patients included in this study. In 158 cases, clot length could not be determined with CTA and instead was estimated using NCCT. Hospital A used 1 mm slice thickness on multiphase CTA for clot length measurements (see Supplementary Figure 1). Hospital B and C performed CTA using 0.6 mm slice thickness. In 6 cases multiple occlusive thrombi were detected and measured on CTA/NCCT. In these cases, the sum of the lengths was used as overall clot length.

After thrombectomy, the extracted clots were collected per pass separately, placed in 10% formalin in separate pots and shipped to NUI Galway, where ECA was calculated. The thrombus measurements were made by a researcher who was blind as to the angiographic outcome to avoid any kind of unconscious bias. Upon arrival, a gross photograph of each thrombus was taken using a Canon EOS 1300 D camera. For the gross photo, all thrombus fragments collected in the same pot were placed on a formalin absorbent pad and a ruler that had both cm and mm was also included in the visual field. The camera was placed on a tripod and the distance between the object and the lens of the camera was set at 10 cm. Area measurements to calculate ECA were made using ImageJ software. After having opened the photograph in ImageJ and having set the scale, the polygon tool was used to draw a region of interest around a fragment of the clot, and the area of that fragment was measured (see Supplementary Figure 2). For cases involving multiple clot fragments, we summed the area measurements of all fragments.

### Statistical analysis

IBM SPSS-25 software was used for statistical analysis. Quantitative variables did not follow a standard normal distribution (as indicated by the Kolmogorov-Smirnov test and Shapiro-Wilk). The non-parametric Spearman’s correlation coefficient test was used to evaluate the correlation between paired data (rho). Kruskal-Wallis test was used to assess statistically significant difference among the groups (H-value). Chi-square test was used to assess an association between size of extracted clots and final recanalization outcome. A level of statistical significance for all analyses was set at p < 0.05 (two-sided). Results are reported as median [IQ1-IQ3] or number and % of cases.

## Results

### Baseline characteristics

Of the 500 patients included in this study, Intravenous thrombolytics (IV rtPA) were administered to 218 patients (43.6%), while 282 (56.4%) were treated with mechanical thrombectomy alone. Successful reperfusion, defined as mTICI ≥2 b, was achieved in 92.8% of patients. This high level of successful reperfusion reflects the fact that only patients for whom at least some thrombus material was recovered through thrombectomy were included in this study. The average clot length on CTA/NCCT was 12 mm, and the average ECA was 39mm^2^. Baseline characteristics of the patient cohort are summarized in Table 1.

**Table 1.** Baseline characteristics of the patients.

	Whole patient cohort (N = 500)	rtPA Yes patients (N = 218)	rtPA No patients (N = 282)
Clot length on NCCT/CTA (mm)	12.0 [8.0–20.0]	12.75 [8.0–20.0]	11.0 [7.50–20.0]
Extracted clot area (mm^2^)	38.50 [20.70–71.90]	37.15 [17.20–67.40]	39.20 [21.70–84.90]
Single occlusion	N = 374 MCA 276 M1 212 (73.8%) M2 52 M3 3 MCA (not specified) 9 ICA&ICA t 61 (16.3%) VB 33 (8.8%) P1 2 (0.5%) A3 1 (0.3%) CCA 1 (0.3%)	N = 162 MCA 127 M1 98 (78.4%) M2 19 M3 2 MCA (not specified) 8 ICA&ICA t 21 (13.0%) VB 13 (8.0%) P1 1 (0.6%)	N = 212 MCA 149 M1 114 (70.3%) M2 33 M3 1 MCA (not specified) 1 ICA&ICA t 40 (18.9%) VB 20 (9.4%) P1 1 (0.5%) A3 1 (0.5%) CCA 1 (0.5%)
Multiple occlusion	N = 126 Tandem 46 (36.5%) Other dual 49 (38.9%) 3 or more 31 (24.6%)	N = 56 Tandem 21 (37.5%) Other dual 22 (39.3%) 3 or more 13 (23.2%)	N = 70 Tandem 25 (35.7%) Other dual 27 (38.6%) 3 or more 18 (25.7%)
Median number of passes	2 [1–3]	2 [1–3]	2 [1–4]
Final mTICI Score			
mTICI 0	5 (1.0%)	1 (0.5%)	4 (1.4%)
mTICI 1	8 (1.6%)	6 (2.8%)	2 (0.7%)
mTICI 2a	23 (4.6%)	13 (6%)	10 (3.5%)
mTICI 2 b	136 (27.2%)	56 (25.7%)	80 (28.4%)
mTICI 2c	111 (22.2%)	47 (21.6%)	64 (22.7%)
mTICI 3	217 (43.4%)	95 (43.6%)	122 (43.3%)

A3: segment 3 of anterior cerebral artery; CCA: common carotid artery; ICA & ICA t: internal carotid artery and terminus; MCA: middle cerebral artery, M1, M2 & M3 segments/branches of the middle cerebral artery; P1: segment 1 of posterior cerebral artery; VB: vertebrobasilar artery; rtPA: recombinant tissue-plasminogen activator; mTICI score: modified thrombolysis in cerebral infarction (TICI) score; CTA: computed tomography angiography; NCCT: non-contrast computed tomography.

Note: Data given as median [IQ1, IQ3] or N(%) of cases.

### Clot length on CTA/NCCT correlates with extracted clot area

Different slice thickness was used across the hospitals, but a good correlation between clot length on CTA/NCCT and Extracted Clot Area was observed in all cases. In 197 cases multiphase CTA was used at 1 mm thickness and the correlation with ECA was r = 0.562, p < 0.0001*. In 145 cases CTA at 0.6 mm thickness was used and the correlation with ECA was r = 0.579, p < 0.0001*. In 158 cases NCCT at 0.6 mm was used and the correlation with ECA was r = 0.754, p < 0.0001*.

Combining all the measurements, we obtained a strong statistically significant positive correlation between clot lengths on CTA/NCCT and ECA (Spearman’s rho = 0.619, N = 500, p < 0.0001*). When we split the whole cohort into two populations, based on whether the patients had been pre-treated with IV tPA or not, the positive correlation between the two variables was slightly stronger for the patients that underwent mechanical thrombectomy alone (Spearman’s rho = 0.694, N = 282, p < 0.0001*) than the patients that received IV thrombolysis before the endovascular treatment (Spearman’s rho = 0.520, N = 218, p < 0.0001*).

In Table 2 the correlation between clot lengths on CTA/NCCT and ECA for the most commonly occurring single vessel occlusions in our patient cohort is reported. Both clot length on CTA/NCCT and ECA are significantly different for the three selected occlusion locations. Unsurprisingly, M2 occlusions are the shortest and the smallest, followed by M1 occlusions and ICA/ICA terminus occlusions are the longest and the largest. The significant positive correlation between clot length on CTA/NCCT and ECA is maintained irrespective of thrombus location.

**Table 2.** Correlation between clot length on CTA/NCCT and Extracted Clot Area in cases with singular occlusion of ICA, M1 or M2.

Occluded vessel	Clot Length on CTA/NCCT (mm)	ECA (mm^2^)	Estimated length of the extracted clot (mm)^a^	Correlation length on CTA/NCCT and ECA
ICA & ICA t	20.0 [14.2–21.5]	111.0 [53.0–149.0]	22.2 [10.6–29.8]	N = 61, r = 0.393, p = 0.002*
M1	11.0 [8.0–15.0]	34.0 [22.0–50.0]	11.0 [7.1–10.0]	N = 212, r = 0.444, p < 0.0001*
M2	6.0 [4.5–8.0]	15 [11.0–21.0]	6.3 [4.6–8.8]	N = 52, r = 0.316, p = 0.017*
Statistical analysis	N = 325, H2 = 98.590, p < 0.0001*	N = 325, H2 = 106.614, p < 0.0001*		

CTA: computed tomography angiography; NCCT: non-contrast computed tomography; M1, M2: segments/branches of middle cerebral artery; ICA & ICA t: internal carotid artery and terminus.

a The measures were obtained by dividing ECA by the average diameter of the occluded vessel as reported by Rai et al.^9^ The results are expressed as median [IQ1-IQ3].

Note: Post-hoc analysis for Length on CTA/NCCTT: M1-M2 p < 0.0001*, M1-ICA&ICA terminus p < 0.0001*, M2-ICA&ICA terminus p < 0.0001*; Post-hoc analysis for Length: M1-M2 p < 0.0001*, M1-ICA&ICA terminus p < 0.0001*, M2-ICA&ICA terminus p < 0.0001*. The correlation coefficient (r) is evaluated as Spearman’s rho.

A direct comparison of length of a clot pre and post thrombectomy is complicated by the impact of thrombectomy on the physical shape of the clot. Dividing the ECA by average vessel diameter,^9^ we have estimated clot length of clots extracted from ICA, M1 and M2 (Table 2). The estimated extracted clot length is very similar to the length measured on CTA/NCCT.

### Thrombus size, number of passes and recanalization outcome

A higher number of passes was associated with a worse recanalization outcome (Table 3). We also found that the total number of passes required for clot removal was significantly associated with both clot length on CTA/NCCT and ECA (Table 4). Larger sized clots required significantly more passes to be retrieved. However, we did not find any statistically significant main effect of clot length on CTA/NCCT or ECA on final mTICI Score (Table 5). We found similar results whether thrombolysis was administered or not (Table 5). To further analyse the relationship between clot size, revascularisation outcome and number of passes required to remove the clot, we grouped the clot size as small, medium or large (Table 6). There was no significant association between length on CTA/NCCT and recanalization outcome when clots were grouped by small, medium or large size (N = 500, *X^2^*=1.197, p = 0.550), however, we observed a significant relationship between ECA and mTICI (Table 6). Medium ECA (20–40 mm^2^) clots are associated with the best recanalisation outcome and require the lowest number of passes for removal (Table 6). This observation was true for the whole cohort and in the subpopulations treated with or without rtPA in addition to thrombectomy.

**Table 3.** Association between higher number of passes required for clot removal and poorer final recanalization outcome.

Final mTICI score	N of passes Overall population (N = 500)	N of passes tPA No (N = 282)	N of passes tPA Yes (N = 218)
mTICI 0	5 [3–6]	6 [4–7]	3 [3–3]
mTICI 1	5 [3–6]	6 [5–6]	4 [2–5]
mTICI 2a	4 [2–5]	5 [4–5]	4 [2–5]
mTICI 2 b	2 [1–4]	3 [1–4]	2 [1–3]
mTICI 2c	2 [1–3]	2 [1–3]	2 [1–3]
mTICI 3	1 [1–2]	1 [1–2]	1 [1–2]
*Statistical analysis*	H5 = 73.134, N = 500, P < 0.0001	H5 = 52.336, N = 282, P < 0.0001	H5 = 23.735, N = 218, P < 0.0001

Note: Data are reported as median [IQ1-IQ3].

**Table 4.** Larger thrombus size is associated with higher number of passes for clot removal.

N of Passes	Clot length on CTA/NCCT (mm)	*Statistical analysis*	Extracted clot area (mm^2^)	*Statistical analysis*
1	10.0 [6.50–15.0]	N = 500, H4 = 31.4, P < 0.0001	32.65 [18.05–49.75]	N = 500, H4 = 50.219, P < 0.0001
2	12.0 [8.50–20.0]		35.80 [21.10–61.00]	
3	15.0 [10.0–21.0]		68.70 [32.30–110.90]	
4	13.0 [7.50–20.0]		45.90 [18.80–122.60]	
5+	16.0 [10.0–22.50]		87.90 [28.40–234.80]	

CTA: computed tomography angiography; NCCT: non-contrast computed tomography.

Note: Data are given as median [IQ1-IQ3].

**Table 5.** Thrombus size is not a strong predictor of final mTICI score.

mTICI Score	Clot length on CTA/NCCT (mm)	*Statistical analysis*	Extracted clot area (mm^2^)	*Statistical analysis*
mTICI 0	21.0 [15.0–51.0]	Total cohort: N = 500, H5 = 7.010, P = 0.220 With thrombolysis: N = 218, H5 = 4.690,p = 0.455 Without thrombolysis: N = 282, H5 = 4.280, p = 0.510	28.4 [10.80–50.10]	Total cohort: N = 500, H5 = 5.858, P = 0.320 With thrombolysis: N = 218, H5 = 2.295, p = 0.807 Without thrombolysis: N = 282, H5 = 6.154, p = 0.292
mTICI 1	10.5 [5.75–12.50]		14.65 [9.20–89.65]	
mTICI 2a	10.0 [9.0–15.0]		34.2 [10.70–81.20]	
mTICI 2b	13.5 [8.0–20.0]		44.95 [18.25–95.40]	
mTICI 2c	12.0 [8.0–20.0]		40.9 [22.00–63.30]	
mTICI 3	11.0 [8.0–18.0]		36.8 [22.80–61.80]	

mTICI Score: modified thrombolysis in cerebral infarction (TICI) score; CTA: computed tomography angiography; NCCT: non-contrast computed tomography.

Note: Data are given as median [IQ1-IQ3].

**Table 6.** Grouping clot ECA as small, medium or large revealed a significant effect of clot size on number of passes and revascularisation outcome, with medium sized clots easiest to remove and with best revascularisation outcome.

Extracted clot area (mm^2^)	≤20	20< and <40	≥40
Number of passes	1 [1–3]	1 [1–2]	2 [1–4]
Number of cases	117	141	242
mTICI 0-2 b	53 (45%)	31 (22%)	88 (36%)
mTICI 2c-3	64 (55%)	110 (78%)	154 (64%)
*Statistical analysis*	N = 500, *χ*^2^=16.202, p < 0.0001*		

Note: Data given as Median [IQ1-IQ3] or N (%) of cases. Chi square (*χ*^2^) statistical analysis.

## Discussion

Our paper demonstrates the strong positive correlation between clot length using CTA/NCCT in advance of thrombectomy and area of extracted clot following thrombectomy, regardless of differences in imaging practice across the three-stroke centres in this study. A non-contrast computed tomography (NCCT) examination is used to exclude or confirm the presence of haemorrhage, but is also helpful to inform on occlusion location, approximate length of occlusion^10^ and, potentially, it can provide some information on clot composition^11^ and stroke etiology.^12^ However, NCCT gives only a unidimensional indication of thrombus size and data generated may vary depending on slice thickness. Some studies reported that conventional NCCT (using 5- or 10-mm slice thickness) is limited in displaying occlusive thrombi in intracranial arteries with a diameter <3 mm and a CT slice-thickness above this value may impair the hyperdense artery sign sensitivity, especially in smaller arteries.^13,14^ At the same time, thin-section NCCT is more sensitive, reliable, and accurate.^15^ Computed tomography angiography (CTA) provides valuable information on the status of large cervical and intracranial arteries and can highlight arterial dissection. CTA is useful to determine clot length, grade collateral blood flow and it can detect thrombosis of the vertebrobasilar system more clearly than NCCT^16^ and is considered one of the most accurate means of localizing an occlusive thrombus. It has previously been suggested that CTA may overestimate clot length compared with NCCT measurements.^17^ Our findings suggest that both methodologies can reliably approximate clot length in advance of endovascular treatment.

The analysis of a sub-group of patients in our cohort having a singular occlusion in the anterior circulation shows that the size of the occluded vessel influences the size of clot, which was already observed in a previous study.^18^ In every case, however, the significant positive correlation between estimate length on CTA/NCCT and relevant ECA was maintained.

It is clear from this study that larger clots may require more passes for removal. It is also clear from this study that there is a relationship between good revascularisation outcome and low number of passes. However, there is a less clear relationship between clot size and mTICI, which is worthy of further study. In the present study, medium sized clots were associated with better recanalization outcomes and lower number of passes than larger clots, which is unsurprising. However, our findings also show that smaller clots were not associated with better recanalization outcome or lower number of passes, which could be related to clot properties and composition, since it has been demonstrated that small, difficult to remove clots are more platelet rich and contain more highly organised fibrin regions.^19,20^

It has been demonstrated that administration of rtPA before mechanical thrombectomy reduces clot size.^21^ The present study demonstrates the correlation between clot length on CTA/NCCT and area of extracted clot was slightly stronger for the cohort of patients that directly underwent mechanical thrombectomy compared to the patients pre-treated with rtPA. This may reflect the continued dissolution of part of the clot, leading to smaller ECA as a consequence of rtPA treatment.

Several studies have observed that recanalization with IV rtPA depends on clot size.^22–26^ There is still much debate about the benefits of rtPA administration prior to endovascular therapy. Potential advantages, such as earlier recanalization, fewer required passes, improved microvascular reperfusion and lysis of distal emboli are counterbalanced by potential disadvantages such as haemorrhagic complications, delay to endovascular treatment and neurotoxicity of alteplase itself. A recent clinical trial demonstrated no difference in clinical outcome between patients undergoing mechanical thrombectomy alone and patients pre-treated with IV rtPA before the endovascular procedure.^27^ Whether clots are more prone to fragmentation with tPA is an interesting question, worthy of further study.

## Strengths and limitations

The main strength of this prospective study is the large patient population arising from three dedicated stroke centres in Europe with a high level of expertise in stroke and interventional treatment.

Limitations of the study include the slight differences in imaging protocols followed by the participating stroke centres and the lack of information on degree of collaterals on CTA/NCCT. Furthermore, the local evaluation of imaging data and revascularisation outcome, rather than evaluation by a core laboratory could also be considered a limitation. However, dividing the analysis per imaging technique used, the results obtained were quite homogeneous.

## Conclusion

The present study has demonstrated evidence of a strong relationship between the evaluation of clot length on CTA/NCCT and the Extracted Clot Area after retrieval by mechanical thrombectomy. This paper also shows that occlusion location influences extracted thrombus size, that bigger clots require more passes for removal, and that poorer revascularisation outcomes occur in cases requiring more passes. The relationship between size and revascularisation outcome is more complex. While the clot size does not seem to have a main effect on final recanalization outcome in patients undergoing mechanical thrombectomy, clots of medium ECA take less passes to remove and are associated with better recanalization outcome than both smaller and larger clots.

## Supplemental Material

sj-pdf-1-eso-10.1177_23969873211024777 - Supplemental material for Correlation between acute ischaemic stroke clot length before mechanical thrombectomy and extracted clot area: Impact of thrombus size on number of passes for clot removal and final recanalization Click here for additional data file. Supplemental material, sj-pdf-1-eso-10.1177_23969873211024777 for Correlation between acute ischaemic stroke clot length before mechanical thrombectomy and extracted clot area: Impact of thrombus size on number of passes for clot removal and final recanalization by Rosanna Rossi, Seán Fitzgerald, Sara M Gil, Oana M Mereuta, Andrew Douglas, Abhay Pandit, Paul Brennan, Sarah Power, Jack Alderson, Alan O’Hare, Michael Gilvarry, Ray McCarthy, Klearchos Psychogios, Georgios Magoufis, Georgios Tsivgoulis, István Szikora, Katarina Jood, Petra Redfors, Annika Nordanstig, Erik Ceder, Turgut Tatlisumak, Alexandros Rentzos, John Thornton and Karen M Doyle in European Stroke Journal

sj-pdf-2-eso-10.1177_23969873211024777 - Supplemental material for Correlation between acute ischaemic stroke clot length before mechanical thrombectomy and extracted clot area: Impact of thrombus size on number of passes for clot removal and final recanalization Click here for additional data file. Supplemental material, sj-pdf-2-eso-10.1177_23969873211024777 for Correlation between acute ischaemic stroke clot length before mechanical thrombectomy and extracted clot area: Impact of thrombus size on number of passes for clot removal and final recanalization by Rosanna Rossi, Seán Fitzgerald, Sara M Gil, Oana M Mereuta, Andrew Douglas, Abhay Pandit, Paul Brennan, Sarah Power, Jack Alderson, Alan O’Hare, Michael Gilvarry, Ray McCarthy, Klearchos Psychogios, Georgios Magoufis, Georgios Tsivgoulis, István Szikora, Katarina Jood, Petra Redfors, Annika Nordanstig, Erik Ceder, Turgut Tatlisumak, Alexandros Rentzos, John Thornton and Karen M Doyle in European Stroke Journal
