# Supplementary material for: Correlation between acute ischaemic stroke clot length before mechanical thrombectomy and extracted clot area: Impact of thrombus size on number of passes for clot removal and final recanalization
Source: Eur Stroke J. 2021 Jul 7;6(3):254–61. doi: 10.1177/23969873211024777 (PMC8564157; doi:10.1177/23969873211024777)
Supplement: sj-pdf-1-eso-10.1177_23969873211024777 - Supplemental material for Correlation between acute ischaemic stroke clot length before mechanical thrombectomy and extracted clot area: Impact of thrombus size on number of passes for clot removal and final recanalization [file sj-pdf-1-eso-10.1177_23969873211024777.pdf]

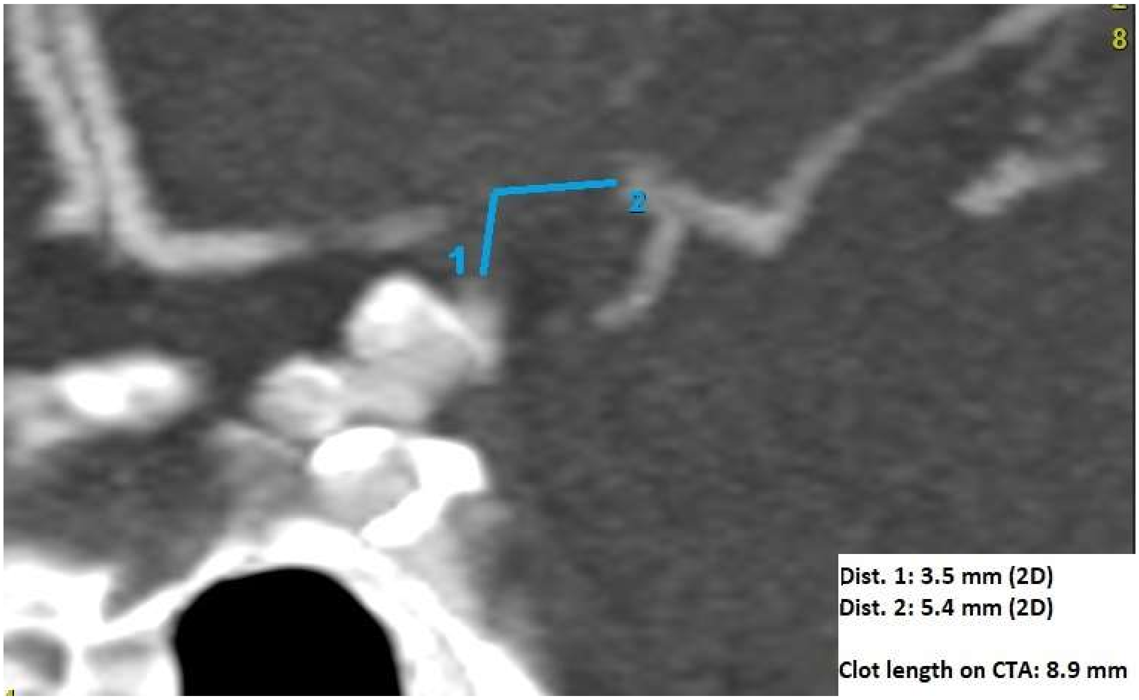

Figure S1: Example of how clot length on CTA is measured. The illustration shows a carotid T occlusion (left side) with good collaterals (retrograde filling all the way down to the distal part of the clot in M1). The measurement was done in 0.6 mm thin slices. Distance 1 and distance 2 are measured separately and the sum of the values is the given clot length on CTA.
