# Supplementary material for: Correlation between acute ischaemic stroke clot length before mechanical thrombectomy and extracted clot area: Impact of thrombus size on number of passes for clot removal and final recanalization
Source: Eur Stroke J. 2021 Jul 7;6(3):254–61. doi: 10.1177/23969873211024777 (PMC8564157; doi:10.1177/23969873211024777)
Supplement: sj-pdf-2-eso-10.1177_23969873211024777 - Supplemental material for Correlation between acute ischaemic stroke clot length before mechanical thrombectomy and extracted clot area: Impact of thrombus size on number of passes for clot removal and final recanalization [file sj-pdf-2-eso-10.1177_23969873211024777.pdf]

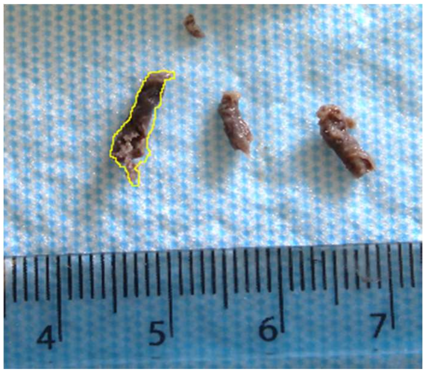

Figure S2: Example of how Extracted Clot Area is measured. A gross photo is taken including a ruler with both mm and cm in the visual field. Using ImageJ software the scale is set and using a drawing tool the area of each fragment is calculated. The final Extracted Clot Area is the sum of the area of all fragments.
